# Immobilization of *Pichia pastoris* cells containing alcohol oxidase activity

**Published:** 2011-12

**Authors:** S Maleknia, H Ahmadi, D Norouzian

**Affiliations:** Pasteur Institute of Iran, Tehran 13164, Iran

**Keywords:** Bioconversion, alcohol oxidase, *P. pastoris*, permeabilization, immobilization

## Abstract

**Background and Objectives:**

The attempts were made to describe the development of a whole cell immobilization of *P. pastoris* by entrapping the cells in polyacrylamide gel beads. The alcohol oxidase activity of the whole cell *Pichia pastoris* was evaluated in comparison with yeast biomass production.

**Materials and Methods:**

Methylotrophic yeast *P. pastoris* was obtained from Collection of Standard Microorganisms, Department of Bacterial Vaccines, Pasteur Institute of Iran (CSMPI). Stock culture was maintained on YPD agar plates****. Alcohol oxidase was strongly induced by addition of 0.5% methanol as the carbon source. The cells were harvested by centrifugation then permeabilized. Finally the cells were immobilized in polyacrylamide gel beads. The activity of alcohol oxidase was determined by method of Tane et al.

**Results:**

At the end of the logarithmic phase of cell culture, the alcohol oxidase activity of the whole cell *P. Pastoris* reached the highest level. In comparison, the alcohol oxidase activity was measured in an immobilized *P. pastoris* when entrapped in polyacrylamide gel beads. The alcohol oxidase activity of cells was induced by addition of 0.5% methanol as the carbon source. The cells were permeabilized by cetyltrimethylammonium bromide (CTAB) and immobilized. CTAB was also found to increase the gel permeability. Alcohol oxidase activity of immobilized cells was then quantitated by ABTS/POD spectrophotometric method at OD _420._ There was a 14% increase in alcohol oxidase activity in immobilized cells as compared with free cells. By addition of 2-butanol as a substrate, the relative activity of alcohol oxidase was significantly higher as compared with other substrates added to the reaction media.

**Conclusion:**

Immobilization of cells could eliminate lengthy and expensive procedures of enzyme separation and purification, protect and stabilize enzyme activity, and perform easy separation of the enzyme from the reaction media.

## INTRODUCTION

Bioconversions are reactions of organic compounds performed by either isolated enzymes or whole cell biocatalysts (1). One of the enzymes in these reactions is alcohol oxidase (alcohol: oxygen oxidoreductase, EC 1.1.3.13, AOX) that is an oligomeric enzyme with eight identical subunits, each containing a non-covalently bound flavine adenine dinucleotide molecule (FAD) as a cofactor. AOX catalyzes the oxidation of primary low molecular weight alcohols into the corresponding aldehydes (2–4). It is produced by methylotrophic yeasts (e.g., *Hansenula*, *Pichia*, and *Candida*) located and assembled in peroxisomes (4). AOX is the first enzyme involved in the methanol oxidation pathway of methylotrophic yeasts and, is able to oxidize other low molecular weight alcohols although its physiological role is the oxidation of methanol (5, 6). AOX is thus responsible for the oxidation of methanol in vivo to the corresponding aldehyde, using molecular oxygen (O2) as the electron acceptor, whereas, it may oxidize many other alcohols in vitro (7). Nowadays, the detection and quantification of alcohols with high sensitivity, selectivity and accuracy is required in many different areas (8, 9). One of the most relevant applications of AOX has been the monitoring of alcohols in the beverages and fermentation industries, clinical chemistry and forensic analysis (4, 10). It is very costly to produce and keep the stability of these enzymes activity constant during the storage process, which make the use of enzymes limited for large-scale industrial process. In a reaction, the stability of enzyme decreases dramatically due to changes in pH, temperature and conformational changes which in turn alter the function of the enzymes. Secondly, since enzymes are soluble, their recovery from a mixture of substrate and product is not economically practical to be reused, rendering the costly enzymatic process even more costly. Therefore, there have been many efforts to recruit immobilized enzymes and cell technology to replace the costly conventional enzymatic process (11, 12). Active immobilization of cells on inert supports facilitates the segregation of cells from the aqueous phase and recovery of cells after the bioreaction is completed. To achieve cell immobilization, entrapment of cells within porous matrices appears to be the most applicable method. The polymers commonly used in such a preparation are including agar, alginate, κ-carrageenan, polyacrylamide, and chitosan (13). Therefore, in the current study, we attempted to describe the development of a whole cell immobilization of *P. pastoris* by entrapment technique using polyacrylamide gel beads in order to measure alcohol oxidase activity and determine alcohol bioconversion.There are some advantages to use yeasts in an immobilization construction including high growth rate, easy manipulation, and ability to grow in a culture media containing a variety of carbon sources. Yeasts are particularly robust with a wide physicochemical tolerance (e.g., pH, temperature, ionic strength, tough cell walls).

## MATERIALS AND METHODS

**Microorganism.** Methylotrophic yeast *P. pastoris* was obtained from Collection of Standard Microorganisms, Department of Bacterial Vaccines, Pasteur Institute of Iran (CSMPI). Stock culture was maintained on YPD agar plates containing 0.25% yeast extract, 0.25% peptone, 5% dextrose and 2% agar (14).

**Chemicals.** All chemicals including 2, 2-Azinobis (3-etilbenzathiazoline-6-sulfonic acid) (ABTS), horseradish peroxidase (HRP), N-cetyl-N,N,N-trimethylammonium bromide (CTAB), Acrylamide, bis-acrylamide, N, N, N’, N’-tetramethylene diamine (TEMED), and Tris-HCl were obtained from Sigma chemical Co.

**Enzyme activity assay.** Yeast cells were grown with shaking in YPMG medium (0.25% yeast extract, 0.25% peptone, 1% malt extract, and 5% dextrose) at 30°C for 24 hours (15). Alcohol oxidase was strongly induced by addition of 0.5% methanol as the carbon source. Cells were harvested by centrifugation, washed three times with 0.1M potassium phosphate buffer, pH 7.5. The cell suspension was then centrifuged at 4000 rpm, 4°C for 15 min. The catalysis of alcohol oxidase in the reaction mixture containing induced yeast cells (0.1 g wet weight), 0.25 ml of 2 mg/ml HRP, 0.1 ml of 2.2 mg/ml ABTS in 100 mM potassium phosphate buffer, pH 7.5, and 2 ml of 17% methanol in the same buffer was monitored routinely by spectrophotometery assay at 420 nm using coupled assays with 2,2′-azinobis-(3-ethylbenzthiazoline-6-sulphonic acid) (ABTS) and peroxidase as described by Tani *et al*. (16). The reaction was started by the addition of methanol to the test cuvette, whereas, in the reference cuvette methanol was replaced by potassium phosphate buffer, pH 7.5. One unit of alcohol oxidase was defined as the amount of enzyme that induces the oxidation of 1 µM of ABTS per minute under the above experimental conditions.

**Permeabilization of*****P. pastoris*******. The cultivated cell suspension which induced by 0.5% methanol as the carbon source, was suspended in sterile potassium phosphate buffer, pH 7.5, containing 0.2% (w/v) CTAB. The suspension was then incubated on a rotary shaker at 150 rpm, 25°C for 30 min. The cells were collected by centrifugation at 4000 rpm at 4°C for 20 min. Treated cells were washed several times with sterile buffer and were stored at 4°C (1).

**Immobilization of*****P. pastoris*******. In a typical procedure, 200 mg wet weight of permeabilized yeast cells was suspended in 6 ml of sterile Tris-HCl buffer, pH 7.5, containing 180 mg acryamide and 5 mg bis-acrylamide in a glass vial. The content was aspirated in a 10 ml syringe and disposed slowly by drops into 50 ml vegetable oil containing 144 mg ammonium persulfate and 144 µl TEMED while swirling gently using a stirrer bar. The polymerization was allowed to take place at room temperature for three hours. Immobilized whole cells beads were separated and washed thoroughly with Tris-HCl buffer. The similarly obtained flat shaped polyacrylamide gel beads were stored at 4°C in Tris-HCl buffer.

**Statistics.** Results are presented as mean ± SD. Statistical analysis used SPSS 18.0 software for one way analysis of variance followed by Student-Newman-Keuls post hoc test. Significant differences were assessed at P < 0.05.

## RESULTS

**Fig. 1:**
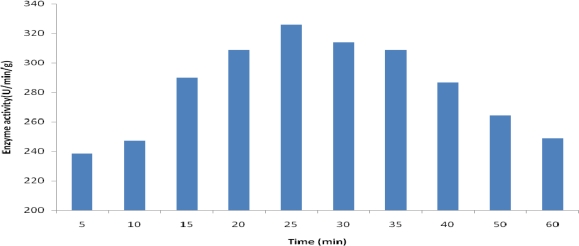
Bioconversion of methanol to formaldehyde in relative different times by using whole cells *P. pastoris*.

In this report we evaluated the oxidation and conversion of methanol to formaldehyde as a bioconversion reaction by employing methylotrophic yeast *P. pastoris* in three different forms such as free whole cells, permeabilized cells and permeabilized cells entrapped in polyacrylamide gel beads. In a time course study, we initially indicated that the alcohol oxidase activity reached the highest level at 25 minutes of incubation period followed by a gradual decrease afterward (Fig.1). Secondly, we evaluated the effect of different concentrations of methanol on bioconversion activity as compared with biomass production.

**Fig. 2:**
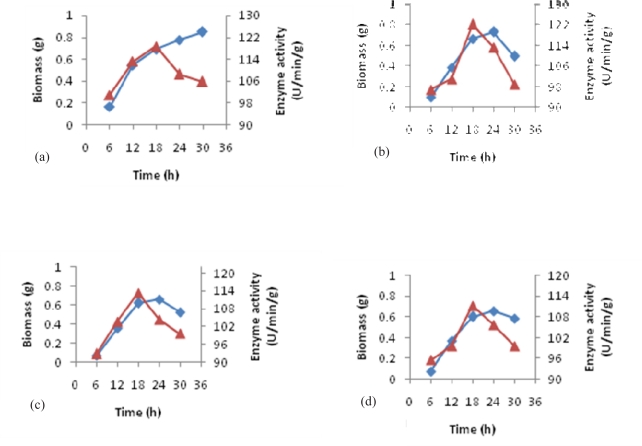
The effect of (a) 0.5%, (b) 1%, (c) 2% and (d) 3% concentration of methanol in whole cells alcohol oxidase activity in comparison with biomass production. (▪) Biomass (g), (▴) Enzyme activity (U/min/g).

Evaluation of alcohol oxidase activity to the highest logarithmic level demonstrated a close relationship with the increase of biomass production. In the mean time, the most bioconversion activity was observed when 1% of methanol was used (Fig. 2b). However, higher concentration of methanol showed less effect on this process, suggesting that any concentration of methanol higher than 1% could have inhibitory effects on bioconversion activity (Fig. 2c and d).

Treatment of whole cells *P. pastoris* with CTAB improved permeabilization of yeast cell membrane and cell wall. This allows the free diffusion of low molecular weight substrates and reaction products increases alcohol oxidase activity of yeast cells. Bioconversion of methanol to formaldehyde in the presence of three different yeast cells types treated with or without CTAB demonstrated that alcohol oxidase activity of permeabilized cells of *P. pastoris* was significantly higher than that in free whole cells and immobilized cells (Table 1). The low alcohol oxidase activity of immobilized cells in comparison with permeablized cells could be due to diffusing restrictions imposed by immobilization that caused the low availability of substrate to the cells.

**Table 1:** Alcohol oxidase activity of three different forms of whole yeast cells.

Type of cell	Mean *	± SD	Enzyme activity (%)
Permeabilized cell	139.17	4.6	100
Free whole cell	75.19	6.4	54.02
Permeabilized immobilized cell	95.13	5.1	68.35

* The mean difference is significant where P < 0.05.There were 4 assays in each experiment.

The relative rates of bioconversion by using different light chain alcohols like ethanol, 1-propanol and 2-butanol were studied as well. It was shown that the relative activity of alcohol oxidase in the immobilized whole cell was significantly higher in the presence of 2-butanol compare to other substrates (Tables 2 and 3).

**Table 2:** Effect of different alcohols on alcohol oxidase activity of permeabilized immobilized *P. pastoris* yeast cells.

Substrate	Mean of Specific Enzyme Activity (U/min/g)	± SD	Relative Activity (%)
Methanol	274.06	29.05	100.00
Ethanol	282.61	27.44	103.12
1-Propanol	285.74	27.10	104.26
2-Butanol	323.17*	34.55	117.92

* The mean difference is significant where P < 0.05.There were 6 assays per substrate.

**Table 3:** Multiple comparisons between different substrates.

(I) Substrate	(J) Substrate	Mean Difference (I-J)	P-value
Methanol	Ethanol	−8.542	.624
	1-Propanol	−11.677	.503
	2-Butanol	−49.107*	.010
Ethanol	Methanol	8.542	.624
	1-Propanol	−3.135	.857
	2-Butanol	−40.565*	.028
1-Propanol	Methanol	11.677	.503
	Ethanol	3.135	.857
	2-Butanol	−37.430*	.041
2-Butanol	Methanol	49.107*	.010
	Ethanol	40.565*	.028
	1-Propanol	37.430*	.041

* The mean difference is significant where P <0.05.There were 6 assays per substrate.

## DISCUSSION

These results indicate that permeabilized immobilized yeast cells increase enzyme activity up to 14% as compared to free cells. The low activity of immobilized cells at lower concentration of methanol could be due to diffusing limitation caused by recondensation of substrate into immobilized cells (17). Barzana et al. (18), Lamara and Legoy (19) and Young and Russel (20) reported the same findings while studying oxidation and diversion of ethanol to acetaldehyde by alcohol dehydrogenase, lipase of *Candida cylindracea*. Norouzian et al (1) also reported the limitation of substrate penetration into the immobilized whole cell *Saccharomyces cerevisiae* for bioconversion of alcohols to aldehydes. The relative activity of alcohol oxidase in immobilized whole cell was at the highest level (323.17 U/min/g) when 2-butanol was employed as a substrate, whereas, other substrates including methanol, ethanol and 1-propanol showed lower alcohol oxidase activity (274.06 U/min/g, 282.61 U/min/g and 285.74 U/ min/g, respectively). In conclusion, immobilization of cells could be used as an appropriate protocol for alcohol biosensors, aldehyde production and other industrial applications. This study demonstrated that immobilization of cells containing specific enzymes has further advantages such as elimination of long and expensive procedures for enzyme separation and purification, protection of enzyme activity and stability, and easy separation from the reaction media to be reused thus yielding decreased in production costs. No attempt has been made so far to use the immobilized whole cells to optimize the process for aldehydes production. However, further studies are required to evaluate more carriers for immobilization of *P. pastoris* which have good mechanical properties and strong diffusibility of substrates. Further developmental studies are being directed in our laboratory toward applying the immobilized whole cells to a bioreactor for the continuous production of aldehydes.
